# Pan-cancer analysis reveals IGFL2 as a potential target for cancer prognosis and immunotherapy

**DOI:** 10.1038/s41598-023-27602-7

**Published:** 2023-04-13

**Authors:** Yuqi Wang, Hongwei Yuan, Genquan Yue, Lingyan Zhao, Yuan Xia, Nan Zhang, Hailing Li, Dongyang Liu, Yubo Su, Haisheng Wang, Yumin Gao

**Affiliations:** 1 School of Public Health, Inner Mongolia Medical University, Hohhot, China; 2 Department of Pathology, School of Basic Medicine, Inner Mongolia Medical University, Hohhot, China; 3 Department of Urology, Affiliated Hospital of Inner Mongolia Medical University, Hohhot, China; 4 Key Laboratory of Molecular Epidemiology of Chronic Diseases, Inner Mongolia Medical University, Hohhot, China; 5 Department of Biochemistry and Molecular Biology, School of Basic Medicine, Inner Mongolia Medical University, Hohhot, China

**Keywords:** Molecular medicine, Prognostic markers, Oncology

## Abstract

*Insulin-like growth factor like family member 2* (*IGFL2*) is a gene in the *IGFL* family, located on chromosome 19, whose role in cancer is unclear, and the aim of this study was to investigate the relevance of *IGFL2* expression, prognosis, immunity, and mutation in pan-cancer. Obtaining information from The Cancer Genome Atlas and The Genotype-Tissue Expression Project (GTEx) databases for expression analysis and combining with The Gene Expression Profile Interaction Analysis database for prognostic aspects. Analysis of immune cell infiltration by TIMER and CIBERSORT algorithms. Calculation of correlation of immune-related genes with *IGFL2* expression and tumor mutational burden and microsatellite instability. Mutations and DNA methylation were analyzed using the cBioPortal database and the UALCAN database, and functional enrichment was performed using Gene set enrichment analysis (GSEA). *IGFL2* expression is significantly elevated in tumor tissue and high expression has a worse prognosis in most cancers. In immune correlation analysis, it was associated with most immune cells and immune-related genes. In most cancers, *IGFL2* methylation is lower and the group with mutations in *IGFL2* has a worse prognosis than the normal group. The GSEA analysis showed that *IGFL2* was significantly enriched in signaling and metabolism. *IGFL2* may be involved in the development of many types of cancer, influencing the course of cancer with different biological functions. It may also be a biomarker for tumor immunotherapy.

## Introduction

Cancer remains a disease with very high morbidity and mortality in epidemiological studies. In 2020, there will be 19.3 million cancer patients and more than 10 million deaths worldwide, with breast cancer being the most common cancer and lung cancer having the highest mortality rate at 18 percent. 28.4 million people will have cancer by 2040^1^. Tumor immunotherapy has been shown to be effective in cancer treatment by using immune cells to eliminate tumor cells^2^.Therefore, predicting biomarkers and identifying tumor treatment targets are crucial in cancer treatment. The human *IGFL* gene encodes a protein of approximately 100 amino acids and contains 11 conserved cysteine residues, including two CC motifs. This family consists of four genes and two pseudogenes, *IGFL1-IGFL4*, *IGFL1P1* and *IGFL1P2*, all clustered on chromosome 19 at 35 kb intervals, which have structural homology with *the insulin-like growth factor* (*IGF*) family^3^. The *IGF* family of genes is a systemic growth factor and a major regulator of cell proliferation, differentiation and apoptosis ^4^. Its dysfunction or dysregulation may destabilize tissues and act on target organs in an autocrine, paracrine and endocrine manner, while activating various intracellular signaling pathways to promote cell proliferation, transformation and inhibit apoptosis, leading to the development of malignant tumors ^5^. *IGF* family members have been shown to play an important role in a variety of tumorigenesis, such as gastric cancer^6^, colorectal cancer^7^, and lung cancer^8^.Among the relevant studies on the IGFL family, IGFL2 is particularly well represented and deserves analysis.Studies on *IGFL2* have found that its expression is upregulated in many cancers, and as a homolog of the *IGF* family, this pattern may be consistent with *IGF* family members. However, the mechanism of *IGFL2* in various carcinogenesis is unclear and there is a lack of correlation analysis of *IGFL2*. Herein, we have comprehensively analyzed the expression, prognosis, immunological and biological roles of *IGFL2* in cancer based on TCGA database data to explore the multifaceted relationship between *IGFL2* and cancer.

## Materials and methods

### Data acquisition & processing

The expression, clinical correlation, and mutation data for a total of 10,534 cases of 33 cancers from The Cancer Genome Atlas (TCGA) (https://portal.gdc.cancer.gov/) database^9, 10^ were obtained from the UCSC browser (http://xena.ucsc.edu/) for basic processing of raw data; and the normal tissue information was supplemented with gene data from The Genotype-Tissue Expression Project (GTEx) (http://gtexportal.org) database^11^ for normal tissues. 33 cancer types were included: Adrenocortical carcinoma (ACC), Bladder Urothelial Carcinoma (BLCA), Breast invasive carcinoma (BRCA), Cervical squamous cell carcinoma and endocervical adenocarcinoma (CESC), Cholangiocarcinoma (CHOL), Colon adenocarcinoma (COAD), Lymphoid Neoplasm Diffuse Large B-cell Lymphoma (DLBC), Esophageal carcinoma (ESCA), Glioblastoma multiforme (GBM), Head and Neck squamous cell carcinoma (HNSC), Kidney Chromophobe (KICH), Kidney renal clear cell carcinoma (KIRC), Kidney renal papillary cell carcinoma (KIRP), Acute Myeloid Leukemia (LAML), Brain Lower Grade Glioma (LGG), Liver hepatocellular carcinoma (LIHC), Lung adenocarcinoma (LUAD), Lung squamous cell carcinoma (LUSC), Mesothelioma (MESO), Ovarian serous cystadenocarcinoma (OV), Pancreatic adenocarcinoma (PAAD), Pheochromocytoma and Paraganglioma (PCPG), Prostate adenocarcinoma (PRAD), Rectum adenocarcinoma (READ), Sarcoma (SARC), Skin Cutaneous Melanoma (SKCM), Stomach adenocarcinoma (STAD), Testicular Germ Cell Tumors (TGCT), Thyroid carcinoma (THCA), Thymoma (THYM), Uterine Corpus Endometrial Carcinoma (UCEC), Uterine Carcinosarcoma (UCS), and Uveal Melanoma (UVM).

### *IGFL2* expression analysis

The expression of *IGFL2* in pan-cancer was analyzed using the Timer2.0 (http://timer.cistrome.org/) online tool^12^ Since there is too little normal tissue in some cancers, the transcriptome RNA-seq data of TCGA and GETx and normal tissue data were log2 transformed simultaneously to match the differential expression information between tumor and normal tissue. It was also plotted with R software to determine the changes in *IGFL2* expression in different cancer types. *p* < 0.05 is statistically significant.

### Clinical data correlation analysis

According to the expression of *IGFL2*, the groups were divided into high and low expression groups by median expression level, and COX regression analysis was performed to investigate the correlation and hazard ratio (HR) between it and the prognosis of different cancers, and the correlation between *IGFL2* and survival was assessed by Kaplan–Meier curve and log-rank test. Forest plots and K-M curves were plotted. In addition, clinical staging of selected tumor patients was analyzed using The Gene Expression Profile Interaction Analysis (GEPIA) (http://gepia.cancer-pku.cn/) database^13^ to investigate whether expression correlated with clinical staging.

### Relationship between *IGFL2* expression and immunity

Cell type identification was performed using TIMER and the CIBERSORT algorithm^14^ to assess the relationship between *IGFL2* expression and 22 immune cell subtypes based on expression files. The most commonly used drugs in immunotherapy target and inhibit the immune checkpoint pathway to help the immune system overcome the immune escape achieved by checkpoint overexpression and reactivate immune predation by neoantigenic cancer cells. Therefore, we performed an analysis of immune checkpoints to explore the relationship of their associated genes and, in addition, assessed the proportion of immune substrate components in the tumor microenvironment (TME) using the ESTIMATEScore^15^. Tumor mutational burden (TMB) and microsatellite instability (MSI) play important roles in the tumor microenvironment^16, 17^, so we evaluated the relationship between *IGFL2* on TMB and MSI.

### DNA methylation and gene mutation correlation analysis

The UALCAN (http://ualcan.path.uab.edu/analysis.html) database^18^ was used to analyze *IGFL2* methylation levels in different tumors and normal tissues, with Student's t-test for difference assessment; The online cBioPortal database (http://www.cbioportal.org/) for cancer genomics was used to obtain gene mutation profiles and prognosis^19^.

### GSEA enrichment analysis

*IGFL2* expression was divided into high and low expression groups and analyzed for significant biological pathways by Gene set enrichment analysis (GSEA) enrichment analysis using the gene set KEGG and the immune-related HALLMARK gene set^20–22^, where gene sets with |NES|> 1, NOM *p* < 0.05 and FDR q < 0.25 were considered to be significantly enriched.

### Statistical analysis

Analyses were all based on R (3.6.3) using R packages including tidyverse, survival, ggplot2, fmsb, limma, estimate, etc. *p* < 0.05 was considered statistically significant.

## Results

### Expression of *IGFL2* in cancer

**Figure 1:**
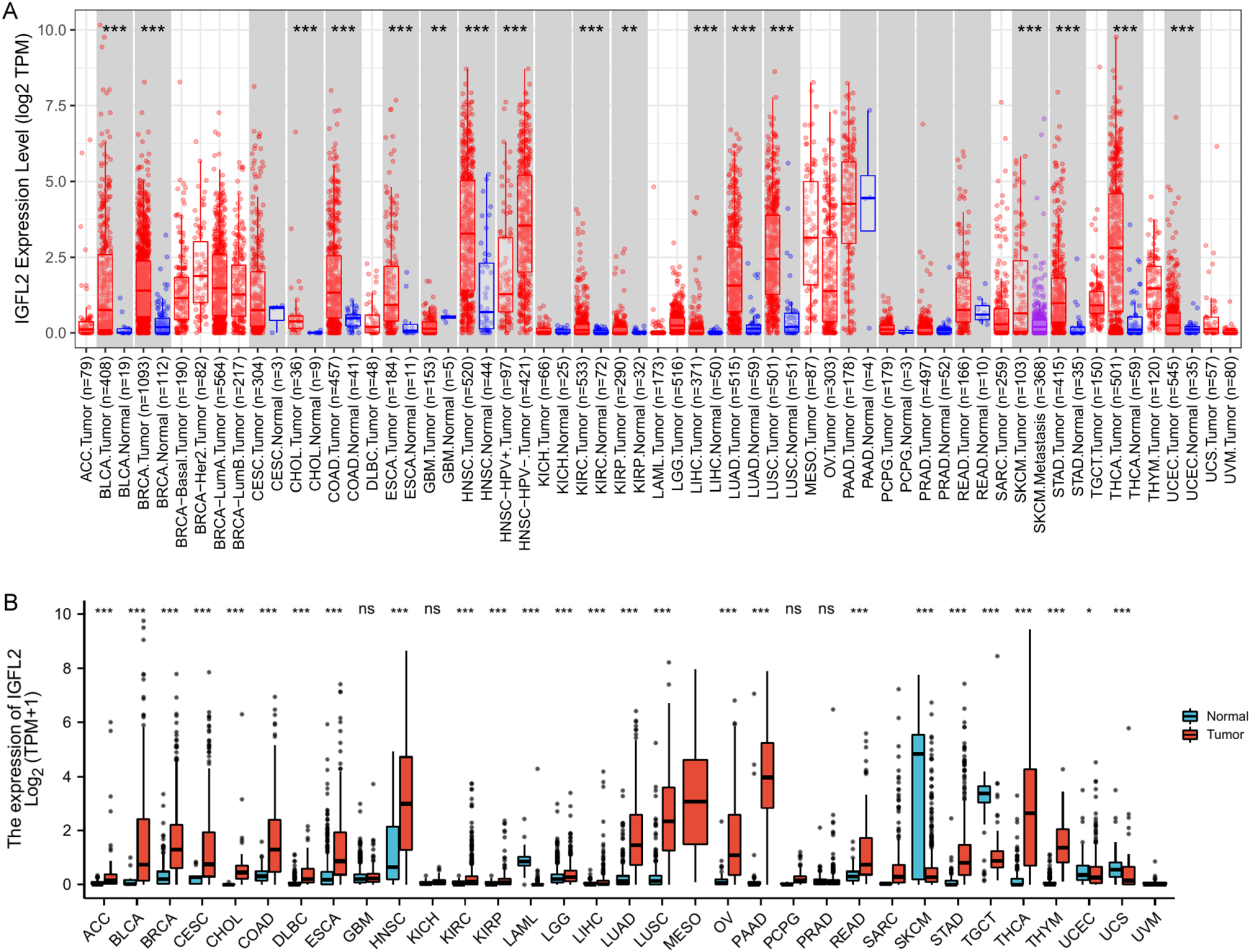
Expression of IGFL2 between normal and tumor tissues (**A**) *IGFL2* expression profiles from TIMER. (**B**) *IGFL2* expression profiles from TCGA and GTEx databases. ***p < 0.001 **p < 0.01 *p < 0.05.

According to the TIMER2.0 database results, the difference in *IGFL2* expression between cancer and normal tissues was significant in most cancers, including BLCA, BRCA, CHOL, COAD, ESCA, GBM, HNSC, KIRC, KIRP, LIHC, LUAD, LUSC, SKCM, HNSC, STAD THCA, and UCEC, while *IGFL2* expression was higher in normal tissues than in cancerous tissues in GBM. Since some cancers lacked normal tissue controls, we obtained basic information from the TCGA database and supplemented the normal tissue controls from the GTEx database, which showed (Fig. 1) that, in addition to the above cancers, the expression of ACC, CESC, DLBC, LAML, LGG, OV, PAAD, READ, TGCT, THYM and UCS differed significantly, suggesting that *IGFL2* may be a participant in oncogenic events. In contrast, *IGFL2* expression was higher in normal than tumor tissues of SKCM, TGCT, UCEC, and UCS.

### The prognostic value of *IGFL2* in cancer

**Figure 2:**
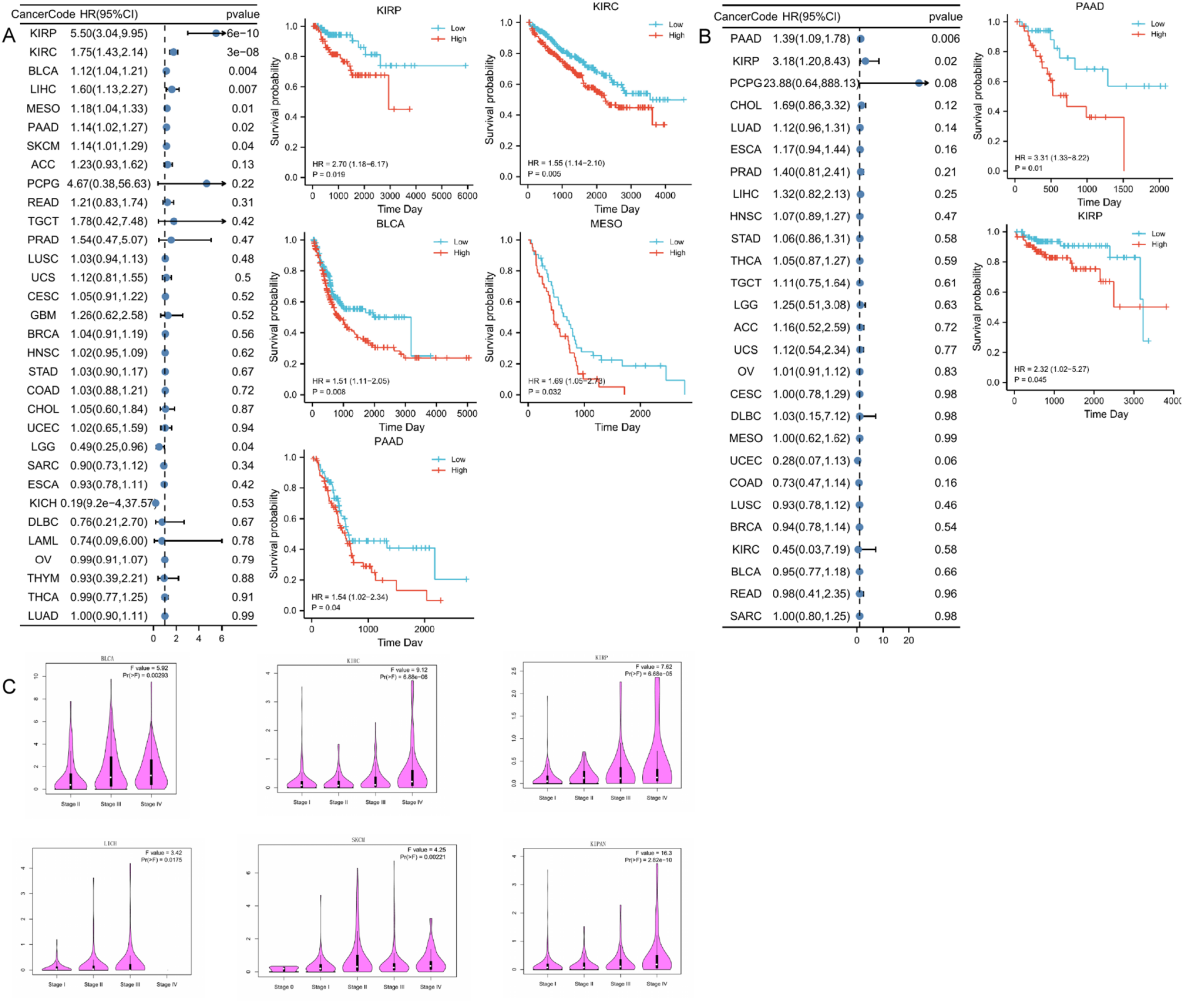
The relationship between *IGFL2* and clinical prognosis. (**A**) Prognosis of *IGFL2* in overall survival. (**B**) Prognosis of *IGFL2* in disease-free survival. (**C**) Positive correlation between *IGFL2* and cancer staging in the GEPIA database.

We comprehensively analyzed the prognosis-related information in pan-cancer based on TCGA clinically relevant information, where survival indicators included overall survival (OS), disease-free survival (DFS). the results of COX regression analysis showed (Fig. 2A) that *IGFL2* was associated with a variety of tumors, including KIRP, KIRC, BLCA, MESO, PAAD and other cancers. And the K-M survival curves showed (Fig. 2A) that the HR and confidence intervals of KIRC,BLCA,KIRP,MESO,PAAD were 1.55 (1.14–2.10), 1.51 (1.11–2.05), 2.70 (1.18–6.17), 1.69 (1.05–2.73), 1.54 (1.02–2.34), respectively, representing that high expression of *IGFL2* was associated with poor prognosis. After changing the survival index to DFS (Fig. 2B), the statistically significant cancers were PAAD, KIRP, and in the K-M survival curve analysis (Fig. 2B), the elevated expression of *IGFL2* in PAAD 3.31 (1.33–8.22) and KIRP 2.32 (1.02–5.27) affected the prognosis. We further analyzed the relationship between *IGFL2* expression and different clinical stages of cancer (stage0-IV) using the GEPIA database, and the results are shown in the figure (Fig. 2C). It can be found that there is a trend of elevated *IGFL2* expression in BLCA, KIRC, KIRP, LICH, and SKCM. Then we analyzed the samples of KIRC, KIRP, and KICH simultaneously to obtain the results of mixed renal carcinoma, which showed that *IGFL2* expression was significantly higher in the advanced stage of cancer than in the early clinical stage, and was a risk factor affecting prognosis.

### Immunocorrelation analysis

#### Analysis of *IGFL2* expression and immune cell infiltration

**Figure 3:**
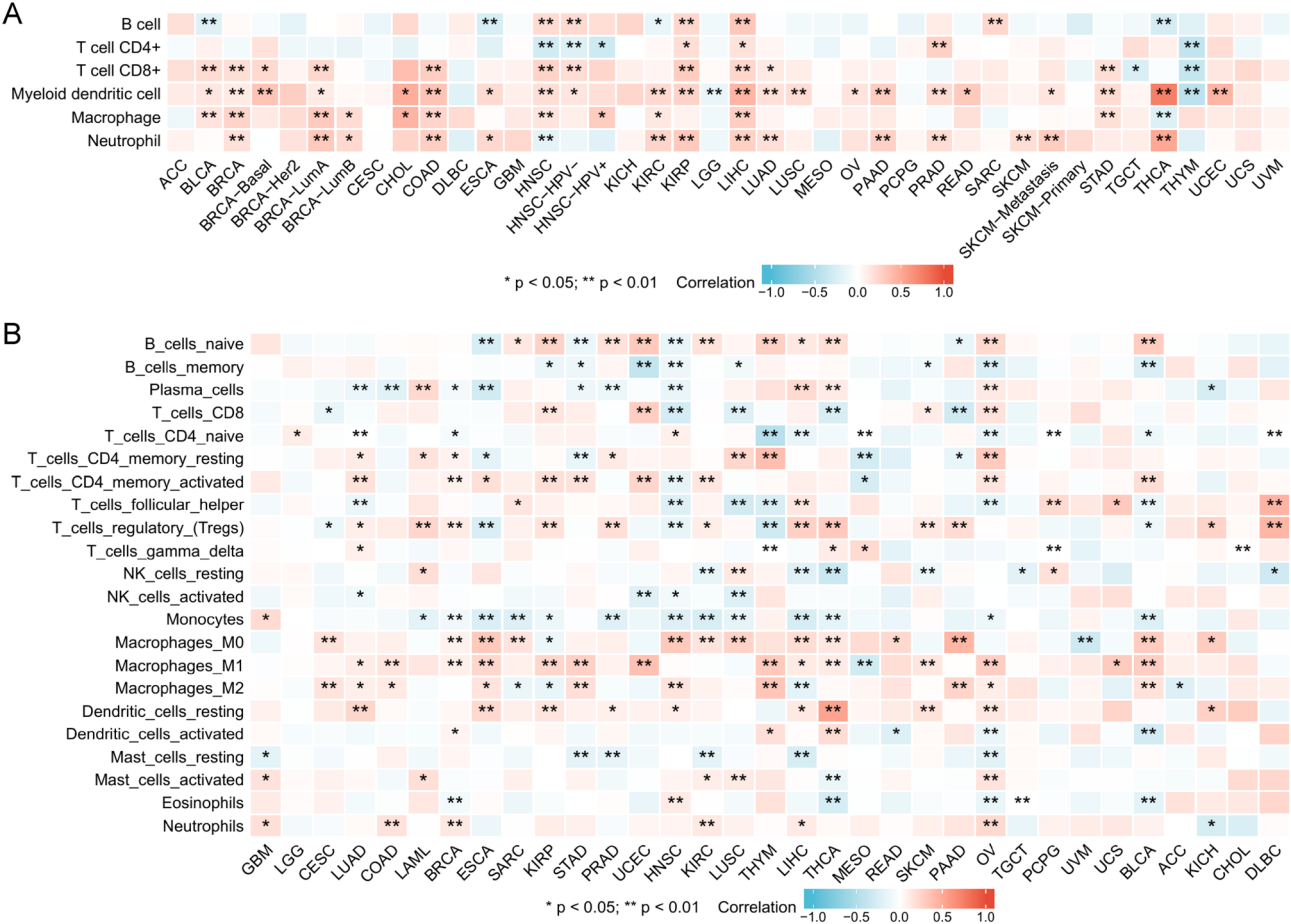
Correlation of *IGFL2* with immune cells. (**A**) Correlation of *IGFL2* expression with 6 immune cell types in the TIMER algorithm. (**B**) Correlation of *IGFL2* with 22 immune cell subtypes in the CIBERSORT algorithm.

Based on the TIMER2.0 database, the relationship between B cells, CD4+ T cells, CD8+ T cells, macrophages, neutrophils, and dendritic cells and *IGFL2* expression was analyzed, and the results are shown in the figure (Fig. 3A). It can be found that *IGFL2* was correlated with most immune cells, B cells, CD4+ T cells, CD8+ T cells, macrophages, neutrophils, and Myeloid dendritic cell were correlated with 9, 7, 13, 12, 15, and 24 cancers, respectively. We further calculated the correlation between *IGFL2* and immune cell subsets using the CIBERSORT algorithm (Fig. 3B), including B cells naive, B cells memory, Plasma cells,T cells CD8,T cells CD4 naive,T cells CD4 memory resting, T cells CD4 memory activated,T cells follicular helper,T cells regulatory (Tregs),T cells gamma delta,NK cells resting,NK cells activated,Monocytes,Macrophages M0,Macrophages M1,Macrophages M2,Dendritic cells resting,Dendritic cells activated,Mast cells resting,Mast cells activated,Eosinophils,Neutrophils. *IGFL2* expression correlated with most immune cells in LUAD, BRCA, HNSC, LIHC, THCA, OV, and BLCA, with mostly negative correlations in HNSC. Among immune cell subgroups, monocytes demonstrated negative correlation with *IGFL2*, including LAML, BRCA, ESCA, SARC, KIRP, PRAD, HNSC, KIRC, LUSC, LIHC, THCA, OV, and BLCA.

#### Analysis of immune-related genes

**Figure 4:**
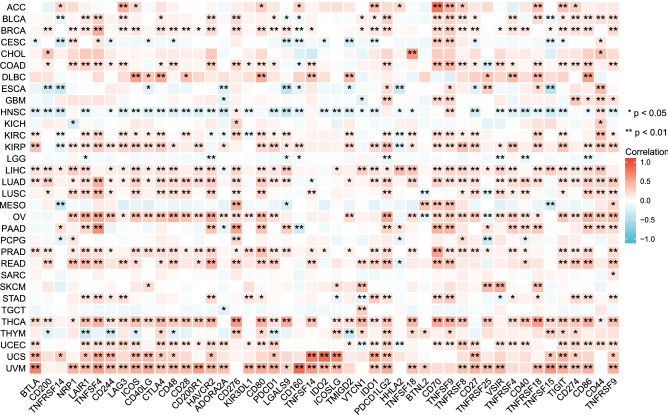
Correlation of *IGFL2* with immune checkpoints.

The importance of immunosurveillance in determining the prognosis of various types of cancer has been widely accepted. Tumors can evade immune responses by exploiting immune checkpoint genes. To further investigate the association between *IGFL2* and the degree of immune infiltration in different cancers, we investigated the correlation between *IGFL2* and immune checkpoint gene expression (Fig. 4). *IGFL2* expression was positively correlated with immune checkpoint genes in almost all cancers, with positive correlations with most immune checkpoints demonstrated in BLCA, BRCA, KIRC, KIRP, LIHC, LUAD, OV, THCA UCEC, UCS and UVM. Furthermore, it is noteworthy that in HNSC, *IGFL2* expression showed a broad negative correlation with immune checkpoint genes. Notably, CD70 correlated relatively strongly with *IGFL2* in ACC, while TNFSF14,IDO2 showed a relatively significant positive correlation with ICOSLG in UCS. Immune checkpoint BTLA appeared positively correlated with CD160 in UVM.

#### Immunosubstrate composition, TMB and MSI analysis

**Figure 5:**
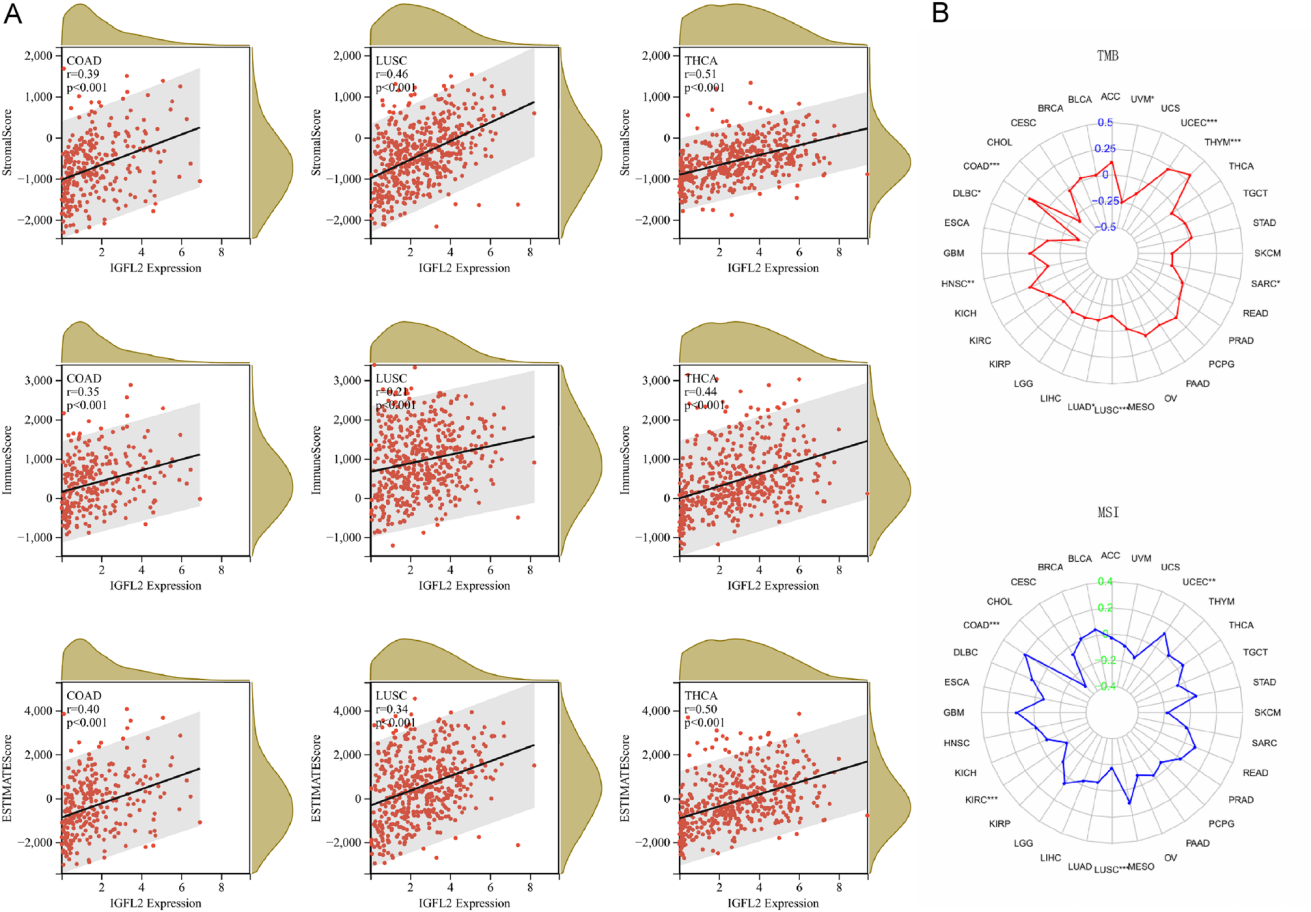
Correlation of scores, TMB and MSI and LCN2 expression in cancer. (**A**) Correlation of ImmuneScore, StromalScore and ESTIMATEScore with *IGFL2* expression in COAD,LUSC,THCA. (**B**) Correlation of *IGFL2* with TMB,MSI. ***p < 0.001 **p < 0.01 *p < 0.05.

The ESTIMATE score allows the calculation of stromal and immune cell ratios in tumor samples to infer tumor purity^15^. We assessed the proportion of immune matrix components using three scores, StromalScore, ImmuneScore and ESTIMATEScore, showed that 24 cancers were positively correlated with *IGFL2* expression, including CHAD (r = 0.39, 0.35, 0.40), LUSC (r = 0.46, 0.21, 0.34), and THCA (r = 0.51, 0.44, 0.50), *p* < 0.001 (Fig. 5A), indicating that *IGFL2* expression was closely associated with the degree of immune infiltration in cancer. In the coming study, we found that tumor mutational load and microsatellite instability have an important influence in the tumor microenvironment, and the assessment of TMB and MSI in tumors has some significance for the analysis of subsequent tumor studies. To explore the influence of *IGFL2* on the tumor microenvironment, we analyzed the correlation between TMB and MSI in pan-cancer (Fig. 5B). In the TMB analysis, there were positive correlations for COAD, THYM, and UCEC, and negative correlations for DLBC, HNSC, LUAD, LUSC, SARC, and UVM. In the MSI analysis, there were positive correlations with COAD and UCEC and negative correlations with KIRC and LUSC.

### Pan-cancer analysis of *IGFL2* methylation levels and genetic alterations

**Figure 6:**
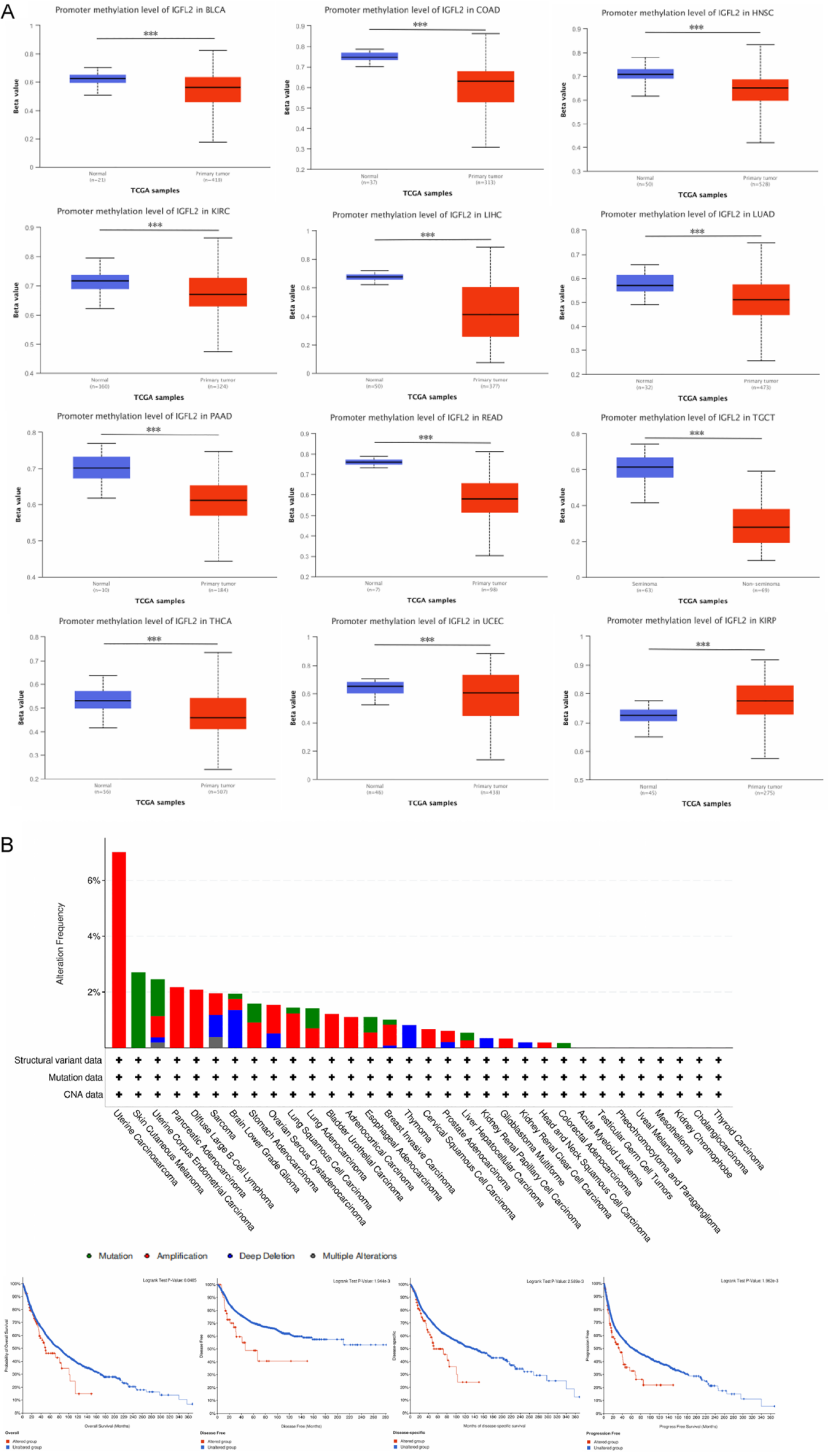
Methylation and mutation of *IGFL2* in relation to cancer. (**A**) Methylation of *IGFL2* in pan-cancer in the UALCAN database. (**B**) Mutation status and mutation prognosis of *IGFL2* in cBioPortal database.***p < 0.001 **p < 0.01 *p < 0.05.

Both hypo- and hypermethylation of DNA contribute to the development of tumors. We investigated the DNA methylation of *IGFL2* using the UALCAN and TCGA databases (Fig. 6A). According to the UALCAN database, significantly lower levels of *IGFL2* methylation were observed in BLCA, COAD, HNSC, KIRC, LIHC, LUAD, PAAD, READ, TGCT, THCA and UCEC tissues compared to normal tissues. And methylation levels were increased in KIRP. Meanwhile, we analyzed the *IGFL2* mutation levels using cBioPortal (TCGA, Pan-Cancer Atlas) (Fig. 6B) and found that the highest mutation levels were found in UCS, over 6%, all of which were gene amplifications. The prognostic impact of mutations was further demonstrated with K-M curves, using OS, DFS, disease-specific survival (DSS) and progression-free survival (PFS) as survival indicators, and dividing the cases into mutation and normal groups, and found that the prognosis of the mutation group was significantly worse in all survival indicators, with statistically significant results (*p* < 0.05). It is suggested that the mutation of *IGFL2* may have influenced the survival rate of cancer.

### Aggregation analysis

**Figure 7:**
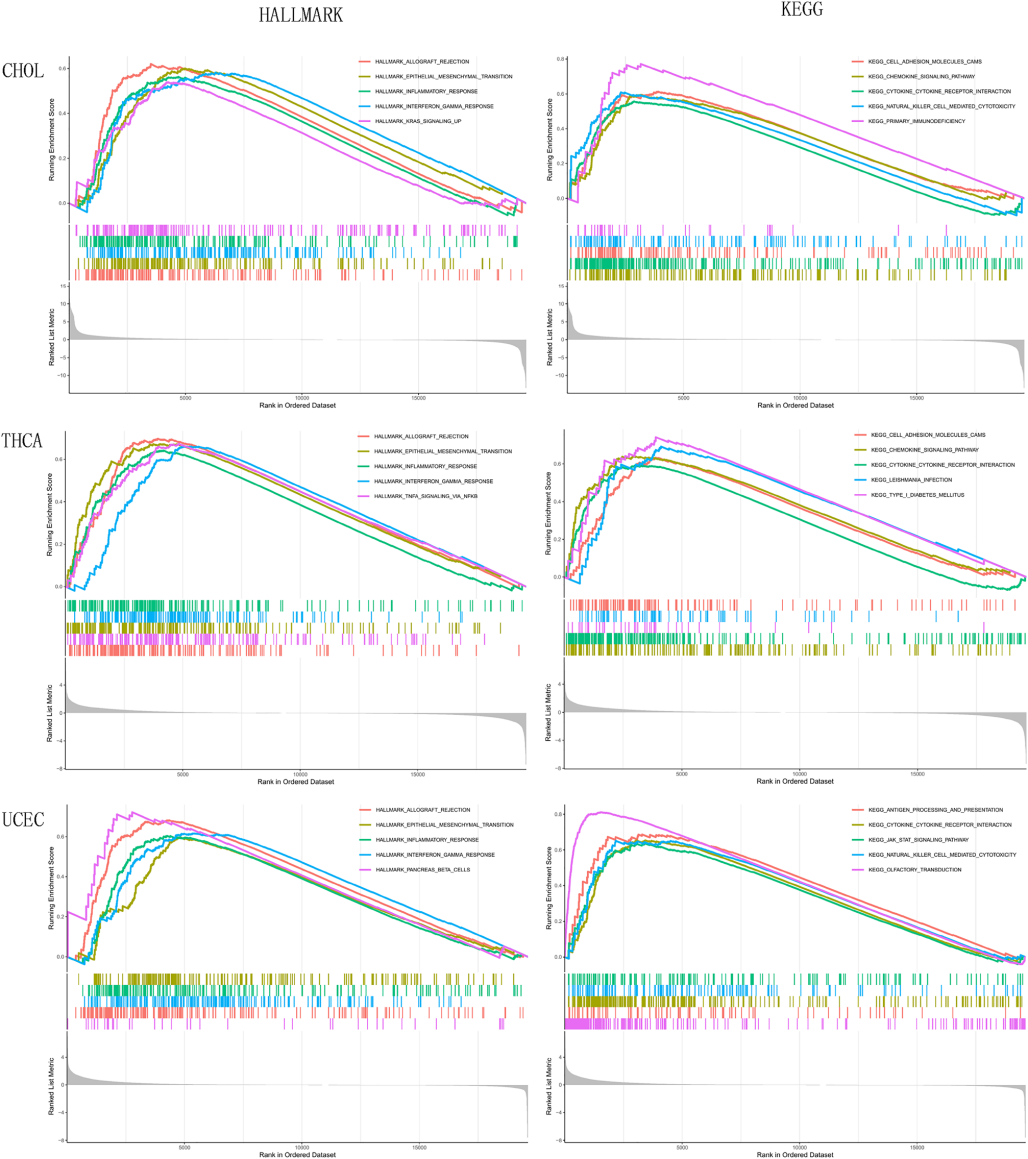
Results of GSEA enrichment analysis. enrichment in CHOL, THCA, UCEC using KEGG and HALLMARK gene sets.

To investigate the potential mechanism of *IGFL2* involvement in carcinogenesis, GSEA was performed to identify the functional enrichment of *IGFL2* high and low expression, and the gene sets KEGG and HALLMARK were used. the KEGG and HALLMARK enrichment items indicated that the high expression of *IGFL2* was mainly related to signaling and metabolism-related activities, including JAK/STAT signaling pathway, inflammatory response, olfactory conduction, etc. (Fig. 7). The results are shown in more detail in the supplementary files (Supplementary Files).

## Discussion

In this study, we comprehensively analyzed the effects of *IGFL2* expression, prognosis, immunity, methylation, mutation and pathways in pan-cancer. In terms of gene expression, *IGFL2* may be widely elevated as an oncogenic molecule in a variety of cancers, while in prognosis-related analysis, high *IGFL2* expression likewise resulted in shorter survival in patients with KIRC,BLCA,KIRP,MESO,PAAD.Notably, in terms of prognosis, *IGFL2* was particularly prominent in urologic tumors (KIRC,KIRP,BLCA), and one study found that insulin-like growth factor-II (*IGF2*) mRNA-binding protein *IMP3* expression was closely associated with clinical grade and prognosis of renal clear cell carcinoma^23^. *IGFL2*, as a homolog of insulin-like growth factor, may be a marker affecting KIRC prognosis.

After establishing that *IGFL2* expression is broadly associated with pan-cancer prognosis, we analyzed whether it influences tumor progression in the tumor microenvironment. Tumor cells, stromal cells, and inflammatory cells in TME suppress lymphocyte and effector cell infiltration, leading to tumor growth^24^ and profoundly affecting tumor prognosis^25^. Tumor progression is accompanied by tumor escape from the immune system^26^, and tumor cells can adopt different strategies to survive and grow, thus limiting the immune system, therefore we comprehensively analyzed the major tumor infiltrating immune cell (TIIC) landscape. This study found that *IGFL2* expression was positively correlated with most immune cells in most cancers. In TIIC analysis, monocytes appeared negatively correlated with IGFL2 in LAML, BRCA, ESCA, SARC, KIRP, PRAD, HNSC, KIRC, LUSC, LIHC, THCA, OV, and BLCA. Monocytes have been found to link innate and adaptive immune responses and can influence TME through various mechanisms, inducing immune tolerance, angiogenesis and increasing tumor cell dissemination^27^. Further collection of more than 40 immune-related genes and analysis of the correlation between *IGFL2* expression and the expression of these genes showed that *IGFL2* was associated with a variety of immune-related genes and showed a positive correlation. The above suggests whether *IGFL2* could be an emerging target for immunotherapy. Interestingly, in immune cell infiltration analysis and immune checkpoint analysis, *IGFL2* showed a negative relationship with most immune cells and immune-related genes in head and neck squamous cell carcinoma, where genes such as PD-L1, PD-L and interferon-γ were reported to have the potential to predict the therapeutic benefit of checkpoint inhibitors^28^, this suggests that *IGFL2* may act on genetic checkpoints to affect cancer. The causes affecting tumor immunity are multifaceted and include factors internal to the tumor and complex interactions between cancer cells and various components of TME. The complexity of TME is also reflected by the stimulatory factors, inhibitory factors, and positive correlations among immune checkpoints in the same group of patients.TMB has now been shown to be a useful biomarker for selective immune checkpoint blockade in a wide range of cancers^16^, and MSI has been confirmed by many authors as a prognostic indicator and a predictor of treatment efficacy^29, 30^, and the results show that *IGFL2* in COAD, THYM, UCEC, DLBC, HNSC, LUAD, LUSC, SARC, UVM may serve as biomarkers for tumor immunotherapy.

Methylation is one of the molecular modifications, methylation of DNA and RNA can regulate the expression of genes involved in the differentiation and function of pro- and anti-cancer immune cells, thus affecting the development of cancer^31^, while abnormal DNA methylation can lead to the development of cancer, hypermethylation in the promoter region can suppress the expression of oncogenes, and reduced DNA methylation occurs in the early stages of tumors, activating proto-oncogenes and leading to the development of cancer^32^. In the analysis of DNA methylation, we found that *IGFL2* was reduced in methylation in a variety of cancers. The possible reason for the appearance of elevated methylation in KIRP is the silencing or inactivation of tumor suppressor genes in cancer cells.Gene mutations are also known to be one of the main causes affecting cancer progression, and our study found that the expected survival in the *IGFL2* mutant group was significantly lower than that in the normal group, again consistent with previous studies.

To explore the biological role of genes in tumorigenesis, we performed GSEA analysis. The long-stranded noncoding RNA of *IGFL2* regulates the Wnt/β-catenin signaling pathway by increasing *SATB1* expression to promote cancer development^33^, and in the results of GSEA enrichment analysis, we identified a significant enrichment of *IGFL2* in the Wnt signaling pathway in thyroid cancer. In contrast, Toll-like receptor signaling, cell adhesion molecules (CAMs), and JAK/STAT signaling pathways all appeared more frequently in GSEA enrichment results, suggesting that *IGFL2* may be involved in related pathways affecting cancer progression.Toll-like receptor signaling is involved in innate and adaptive immune processes and plays an important role in tumorigenesis and progression^34, 35^. CAMs can play a structural role in cell or extracellular matrix adhesion and activate related pathways to enhance cell survival, while the tumor environment leads to proliferation and metastasis of tumor cells due to disruption of CAMs^36^. Aberrant activation of the JAK/STAT signaling pathway has also been demonstrated in a variety of tumors^37, 38^. And in the study of bladder cancer and JAK/STAT signaling pathway, IGF family-related genes were found to activate JAK/STAT signaling pathway to proliferate tumors^39^. In the immune-related HALLMARK set, the pathways of inflammatory response, epithelial mesenchymal transition, and interferon alpha response were enriched.

A recent study on IGFL2 found that miR-802 is a tumor growth suppressor and the long-stranded non-coding RNA of *IGFL2* (*IGFL2-AS1*) can promote the progression of gastric cancer by inhibiting the expression of miR-802^40, 41^, while in breast cancer studies, *IGFL2-AS1* acts as a factor mediating the *KLF5/IGFL1* axis and inhibits miR4795-3p expression thereby affecting breast cancer proliferation^42^. Cen et al. found a significant decrease in proliferation of COAD cells after knocking down *IGFL2-AS1*^43^. In some database-based studies, *IGFL2* was found as a potential pathogenic gene or gene affecting prognosis in liver, breast, renal clear cell, and bladder cancers^44–47^, which is consistent with the results of our analysis.

However, although the present study did a multifaceted pan-cancer analysis of *IGFL2*, it still has some limitations; First, the study was based on data analysis performed in a database, so its causal argument is less powerful than direct experimental studies, and further mechanistic studies would be beneficial to elucidate the role at the molecular and cellular levels of *IGFL2*; Second, there are fewer studies on *IGFL2* and lack of corresponding results to support it.

## Conclusion

Upregulation of *IGFL2* expression was associated with poor patient prognosis and correlated with the level of immune cell infiltration in several cancers. Also, IGFL2 was significantly associated with the expression of immune checkpoint markers, and reduced methylation of IGFL2 was observed in many types of cancers.In conclusion, this study is the first to analyze the multifaceted relationship between *IGFL2* and pan-cancer, and the results of the analysis suggest that *IGFL2* may be a new research direction for tumor therapy.

## Supplementary Information

Supplementary Information.

## Data Availability

Publicly available datasets were analyzed in this study. This data can be found here: The Cancer Genome Atlas (https://portal.gdc.cancer.gov/).

## Abbreviations

ACC: Adrenocortical carcinoma
BLCA: Bladder Urothelial Carcinoma
BRCA: Breast invasive carcinoma
CESC: Cervical squamous cell carcinoma and endocervical adenocarcinoma
CHOL: Cholangiocarcinoma
COAD: Colon adenocarcinoma
DLBC: Lymphoid Neoplasm Diffuse Large B-cell Lymphoma
ESCA: Esophageal carcinoma
GBM: Glioblastoma multiforme
HNSC: Head and Neck squamous cell carcinoma
KICH: Kidney Chromophobe
KIRC: Kidney renal clear cell carcinoma
KIRP: Kidney renal papillary cell carcinoma
LAML: Acute Myeloid Leukemia
LGG: Brain Lower Grade Glioma
LIHC: Liver hepatocellular carcinoma
LUAD: Lung adenocarcinoma
LUSC: Lung squamous cell carcinoma
MESO: Mesothelioma
OV: Ovarian serous cystadenocarcinoma
PAAD: Pancreatic adenocarcinoma
PCPG: Pheochromocytoma and Paraganglioma
PRAD: Prostate adenocarcinoma
READ: Rectum adenocarcinoma
SARC: Sarcoma
STAD: Stomach adenocarcinoma
SKCM: Skin Cutaneous Melanoma
TGCT: Testicular Germ Cell Tumors
THCA: Thyroid carcinoma
THYM: Thymoma
UCEC: Uterine Corpus Endometrial Carcinoma
UCS: Uterine Carcinosarcoma
UVM: Uveal Melanoma
TME: The tumor microenvironment
TMB: Tumor mutational burden
MSI: Microsatellite instability
GSEA: Gene set enrichment analysis
HR: Hazard ratio
CI: Confidence interval
OS: Overall survival
DFS: Disease-free survival
DSS: Disease special survival
PFS: Progression-free survival
TIIC: Tumor infiltrating immune cell
CAMs: Cell adhesion molecules
